# Molecular Dynamics Simulations and Theoretical Model for Engineering Tensile Properties of Single-and Multi-Walled Carbon Nanotubes

**DOI:** 10.3390/nano11030795

**Published:** 2021-03-19

**Authors:** Keiichi Shirasu, Shunsuke Kitayama, Fan Liu, Go Yamamoto, Toshiyuki Hashida

**Affiliations:** 1 Department of Aerospace Engineering, Tohoku University, 6-6-01, Aza-Aoba, Aramaki, Aobaku, Sendai, Miyagi 980-8579, Japan; gyamamoto@tohoku.ac.jp; 2 Fracture and Reliability Research Institute, Tohoku University, 6-6-11, Aza-Aoba, Aramaki, Aobaku, Sendai, Miyagi 980-8579, Japan; shunsuke.kitayama@rift.mech.tohoku.ac.jp (S.K.); fan.liu@rift.mech.tohoku.ac.jp (F.L.); hashida@rift.mech.tohoku.ac.jp (T.H.); 3 School of Mechanical Engineering, Sungkyunkwan University, Suwon 16419, Korea

**Keywords:** carbon nanotube, molecular dynamics simulations, mechanical properties, tensile strength, defect density

## Abstract

To apply carbon nanotubes (CNTs) as reinforcing agents in next-generation composites, it is essential to improve their nominal strength. However, since it is difficult to completely remove the defects, the synthesis guideline for improving nominal strength is still unclear, i.e., the effective strength and the number of nanotube layers required to improve the nominal strength has been undermined. In this study, molecular dynamics simulations were used to elucidate the effects of vacancies on the mechanical properties of CNTs. Additionally, the relationships between the number of layers and effective and nominal strengths of CNTs were discussed theoretically. The presence of extensive vacancies provides a possible explanation for the low nominal strengths obtained in previous experimental measurements of CNTs. This study indicates that the nominal strength can be increased from the experimentally obtained values of 10 GPa to approximately 20 GPa by using six to nine nanotube layers, even if the increase in effective strength of each layer is small. This has advantages over double-walled CNTs, because the effective strength of such CNTs must be approximately 60 GPa to achieve a nominal strength of 20 GPa.

## 1. Introduction

The exceptional mechanical properties of carbon nanotubes (CNTs) make them highly attractive as potential reinforcing components in next-generation composites, such as in hydrogen storage tanks and space elevator cables. For these applications, webs, sheets, and yarns of multi-walled carbon nanotubes (MWCNTs), which are fabricated by directly dry-drawing MWCNTs from drawable MWCNT forests, have been developed [1,2,3,4,5,6,7,8]. These MWCNT forests are synthesized by the thermal chemical vapor deposition (CVD) method [2,3,4]. However, the conventional fabrication techniques cause structural defects in the CNTs, which significantly affect their mechanical properties [9,10,11]. Indeed, the tensile strength of these tubes was reported to be as low as 10 GPa [12]. To enable the development of CNTs with superior mechanical properties, we have previously investigated the structural–mechanical property relationships of MWCNTs based on a theoretical model of their tensile strength. It was also demonstrated that the nominal (engineering) tensile strength, rather than the effective tensile strength, is the key factor in determining mechanical properties when designing CNT-reinforced composites [12].

Molecular dynamics (MD) has been the predominant computational approach for evaluating the mechanical properties and fracture behavior of CNTs [13,14,15,16]. Byrne et al. [14] showed that defective MWCNTs with sp^3^ interwall bonding exhibited strength values exceeding those of single-walled carbon nanotubes (SWCNTs) of the same size. Additionally, they concluded that composites with suitably designed MWCNTs would outperform most SWCNT-reinforced composites. Furthermore, Xiang et al. [17] used machine-learning-assisted, high-throughput MD simulations to investigate the relationship between the structural parameters and mechanical properties (including the nominal strength) of CNTs with Frenkel-pair-type crosslinks. In these studies [14,17], interlayer crosslinking was introduced to a defect-free MWCNT model to evaluate the CNT’s mechanical properties. On the other hand, a number of studies [18,19,20,21] have demonstrated that exposing vacancy-defective graphite to oxygen at elevated temperatures leads to the formation of approximately circular holes in a surface graphene sheet. To remove amorphous carbon, soot, and highly defective nanoparticles, oxidative etching is often employed in purification procedures for CNTs. However, even in high-quality CNTs, the use of oxidative etching may lead to pitting. For low-crystallinity CVD-grown CNTs, hole defects were observed in the outer layer [22], and the Raman intensity ratio was approximately 1 [12], despite the observation of graphene layers by transmission electron microscopy. This suggests that the CVD process introduces a considerable number of vacancies and hole defects. It has already been demonstrated analytically [9,23,24] that a single significant hole is sufficient to dramatically reduce the fracture strength of a CNT.

In our previous study, we focused on interlayer load transfer by crosslinking, and compared a theoretical model with the experimental variation in the nominal strength of MWCNTs [12]. However, the relationships between the pitting density and effective strength of the pitted nanotubes with different diameters, and between the expected nominal strength of the MWCNTs with a different number of nanotube layers and the given effective strength, are not yet well understood. In this study, the relationships between the pitting density and the effective and nominal strengths of SWCNTs were evaluated using MD simulations. Thereby, the number of layers and effective strength were investigated with the aim of improving the nominal strength of MWCNTs. Thus, following our previous work [12], we are investigating the nanotube structure required to improve the nominal strengths of CNTs.

## 2. Molecular Dynamics Models and Computational Methods

**Figure 1:**
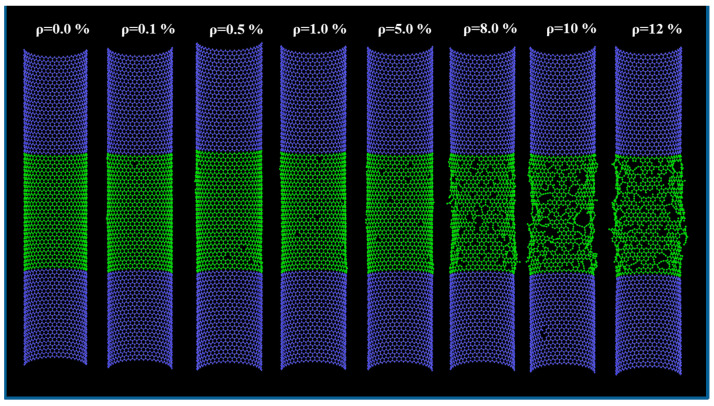
Schematic of computational models with different pitting densities from 0 to 12%.

In this study, various SWCNT models with different diameters (chirality; from (34,0) to (97,0)) were created using BIOVIA Materials Studio (Dassault Systèmes, San Diego, CA, USA). The length of the SWCNTs was fixed at 107.1 Å. The effects of the diameter and pitting density on the effective tensile strength and Young’s modulus of the SWCNTs were investigated using uniaxial tensile loading tests based on the MD method, and processed with open-source software (Large-Scale Atomic/Molecular Massively Parallel Simulator (LAMMPS)) [25]. The adaptive intermolecular reactive empirical bond order (AIREBO) model [26] was used for the MD simulation. Prior to equilibration, the SWCNT models were pitted. The pitting density was in the range of 0–12%, and the pits were randomly introduced into the mobile part of the SWCNT models. To ensure the equilibration of the internal stress, and to minimize the total energy of each model, an isothermal–isobaric (NPT) ensemble was coupled to a Nose–Hoover thermostat, and the relaxation process was conducted under the following conditions: 100 K temperature, zero applied load, and 0.5-fs time step. The pitted SWCNT models with different pitting densities post-relaxation are shown in Figure 1. To avoid the influence of thermal fluctuations, the temperature was reduced to 1 K after the equilibrium period. Based on the experimental parameters presented in previous studies [12,22,27], the uniaxial tensile load was applied along the z-axis to the atoms of the two fixed parts of the SWCNT in the canonical ensembles (NVT). The engineering strain rate was fixed at 3.8 × 10^9^ s^−1^ and a time step at 0.5 fs. Note that the fixed part of the SWCNT (blue atoms shown in Figure 1) also experienced elongation along the tube axis as the load increased. All the atoms in the mobile part, including the middle portion (green atoms in Figure 1), could move freely. To avoid non-physical increases in stress values during tensile loading, the AIREBO potential cutoff distance was modified to 1.79 Å.

For each model, the values of strain and effective stress were obtained. The deformation along the z-axis of the model was divided by the original length to obtain the strain. The stress was determined by dividing the stress tensor by the volume of the carbon atoms, where the stress tensor was obtained using LAMMPS. The effective area comprises the tension loading cross-sectional area, including the wall thickness of the model, as shown in:

(1) $$A_{\mathrm{eff}}=2\pi rt$$

where $A_{\mathrm{eff}}$ is the effective cross-sectional area, *r* is the radius of the nanotube wall, and *t* is the wall thickness (3.4 Å). Young’s modulus was calculated by dividing the effective stress by the strain during elastic stretching. At one pitting density, three independent computations were performed. Thus, we obtained three stress–strain curves for each pitted model, and the Young’s modulus and effective strength were calculated along with the average and standard deviation for each of the pitting densities.

## 3. Results and Discussion

### 3.1. SWCNTs (MD Simulations)

**Figure 2:**
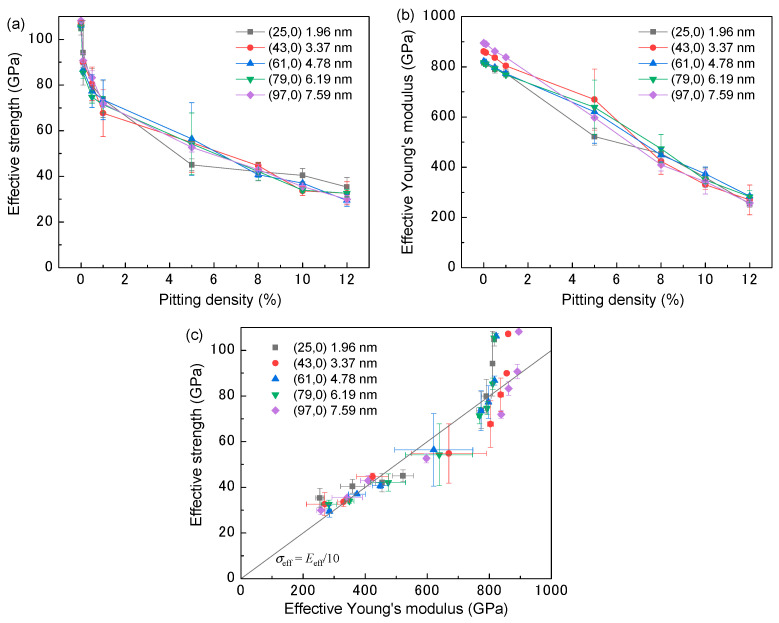
Numerical data indicating trends in (**a**) effective strength and (**b**) Young’s modulus. (**c**) Relationship between effective strength and Young’s modulus. The line in (**c**) indicates ideal strength with respect to the effective modulus, approximately one-tenth of the Young’s modulus.

The tensile strength results for each model are shown in Figure 2. For the intact SWCNTs (pitting density = 0%), the resultant effective strength and Young’s modulus of the AIREBO potential-based tensile test were 107 and 842 GPa, respectively. These values were consistent with those of previous MD simulations [28] and quantum calculations [29]. As seen in Figure 2a, the effective strength declined sharply when the pitting density increased from 0 to 1%. However, the rate of decline slowed above a pitting density of 5%. The effective Young’s modulus declined approximately linearly as the pitting density increased and shows values similar to those of carbon fibers when the pitting density exceeded 10% [30,31]. These relationships between the pitting density and mechanical properties are in reasonable agreement with a previous study [23]. In this model, the effective strength and modulus were independent of the SWCNT diameter. The relationship between the effective strength and modulus is shown in Figure 2c. The solid line in Figure 2c shows the ideal strength with respect to the effective modulus, and is approximately one-tenth of the Young’s modulus [32]. Although the effective strength of the defect-free SWCNT model is higher than this ideal value (i.e., the point is above the solid line in Figure 2c), the pitted models exhibited an effective strength of approximately one-tenth of the Young’s modulus.

In our previous study [12], we evaluated whether effective or nominal strength provided more reliable information on a composite’s load-bearing ability. Assuming that the external forces (*σ*_c_*A*_c_) are applied to the composite in the CNT alignment direction, the following force balance is satisfied:

(2) $$\sigma_{c}A_{c}=\sigma_{\mathrm{eff}}A_{\mathrm{eff}}+\sigma_{m}\left(A_{c}-A_{\mathrm{nom}}\right)$$

where *σ*_c_, *σ*_eff_, and *σ*_m_ are the applied stresses for the composite, CNTs, and matrix, respectively. *A*_c_ is the cross-sectional area of the composite. *A*_eff_ is the effective cross-sectional area of the CNTs, and *A*_nom_ is the full cross-sectional area of the CNTs, including an inner hole. Thus, *A*_eff_ and *A*_nom_ can be written as:

(3) $$A_{\mathrm{eff}}=2\pi t\sum_{k=1}^{n}r_{k}$$

(4) $$A_{\mathrm{nom}}=\pi r_{1}^{2}$$

where $r_{k}$ is the radius of the *k*th layer and *r*_1_ is the radius of the outermost layer. The volume fraction of the CNTs (*V*_f_) is given by:

(5) $$V_{f}=A_{\mathrm{nom}}/A_{c}$$

Thus, the applied stress for the composite can be described by:

(6) $$\sigma_{c}=V_{f}\sigma_{\mathrm{nom}}+\left(1-V_{f}\right)\sigma_{m}$$

**Figure 3:**
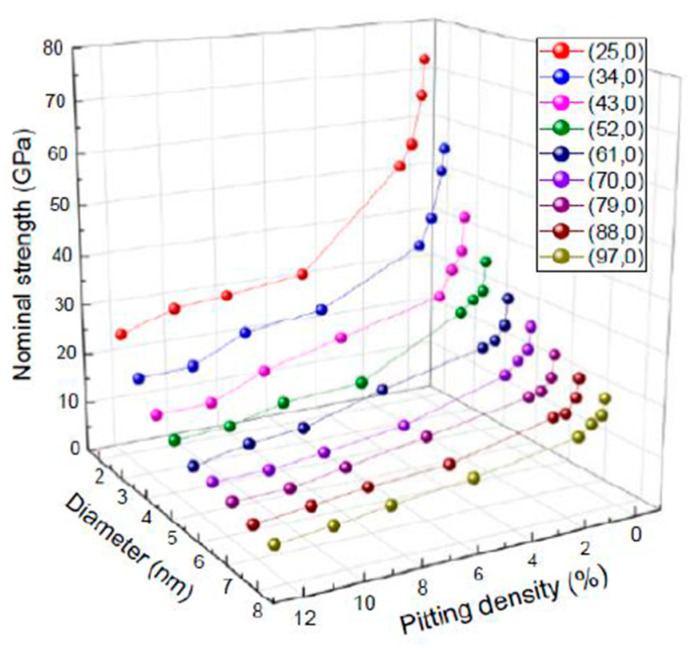
Relationships between carbon nanotube (CNT) diameter, pitting density, and nominal tensile strength of a single-walled CNT (SWCNT).

Here, *σ*_nom_ is the nominal tensile strength. Therefore, an accurate model of the mechanical properties of CNT-reinforced composites should utilize the nominal tensile strength as the index of load bearing. The relationships between the CNT diameter, pitting density, and nominal tensile strength of the SWCNT models are shown in Figure 3. As with the effective strength described previously, the nominal strength decreases with increasing pitting density, and the rate of decrease is especially marked at low defect densities (between 0 and 1%). Additionally, as the SWCNT diameter decreases, the nominal strength tends to increase, owing to the decrease in the cross-sectional area of the hollow core. Nonetheless, the nominal strength of a (25,0) SWCNT (1.96 nm in diameter) with a defect density of 12% was approximately 23 GPa. Our previous study showed that the effective strength of CVD-grown MWCNTs was often below 10 GPa [12]. In some of those MWCNTs, hole defects were observed in the outer layer [22], and the Raman intensity ratio was approximately 1 [12], despite graphene layers being observed via transmission electron microscopy. Therefore, this suggests that the synthesized MWCNTs were composed of nanotube layers with a pitting density above 10%.

### 3.2. MWCNTs (Theoretical Calculations)

The degree of interlayer load transfer can be quantified using the fracture cross-section ratio (*A*_eff_/*A*_nom_), where the higher the fracture cross-section ratio (for a given outer diameter), the larger the number of fractured nanotube layers. Thus, the nominal tensile strength is described by:

(7) $$\sigma_{\mathrm{nom}}=\sigma_{\mathrm{eff}}\frac{A_{\mathrm{eff}}}{A_{\mathrm{nom}}}$$

If it is assumed that perfectly rigid crosslinks between each shell on opposite ends of an MWCNT (arbitrary length) pin each shell together, then the effective tensile strength can be estimated [12]. The applied force is:

(8) $$F=\frac{\sum_{k=1}^{n}r_{k}}{r_{m}}F_{m}$$

where *r*_m_ and *F*_m_ are the radius and internal force of the *m*th layer, respectively. Therefore, the effective tensile strength can be expressed as:

(9) $$\sigma_{\mathrm{eff}}=\frac{F}{A_{\mathrm{eff}}}=\frac{F_{m}}{2\pi r_{m}t}$$

If the outermost layer of the MWCNT is fractured during tensile loading, then Equation (9) indicates that the effective tensile strength is equal to the fracture strength of the outermost shell (*σ*_1_). Furthermore, as shown in Figure 2c, when the Young’s modulus decreases due to the increase in pitting density, the effective strength of the outermost layer is one-tenth of the Young’s modulus. Thus, Equation (7) can be expressed as:

(10) $$\sigma_{\mathrm{nom}}=\sigma_{1}\frac{A_{\mathrm{eff}}}{A_{\mathrm{nom}}}=\frac{E_{\mathrm{eff}}}{10}\frac{2t\sum_{k=1}^{n}r_{k}}{r_{1}^{2}}$$

**Figure 4:**
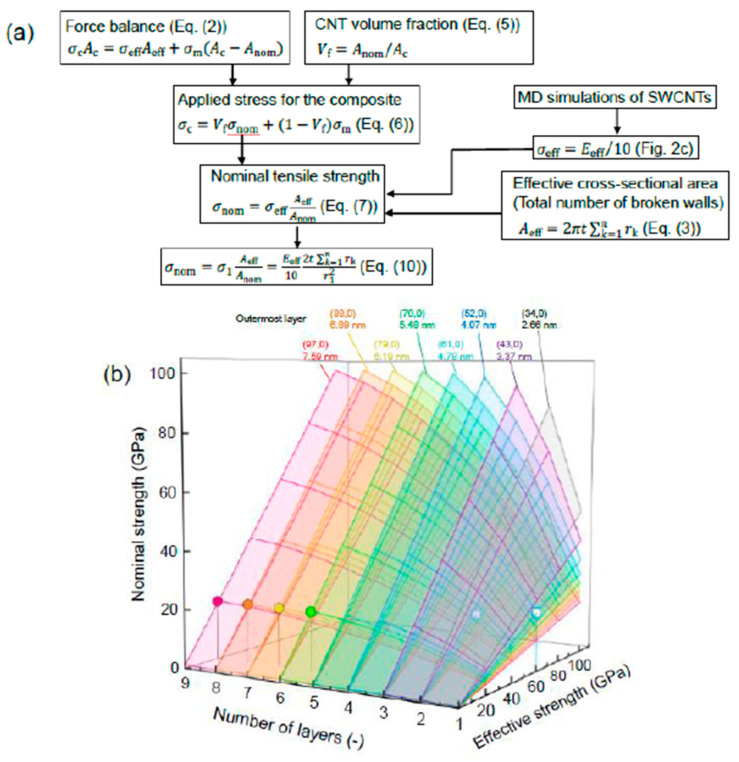
(**a**) Model scheme for nominal strength and (**b**) relationships between number of layers, effective strength of the outermost layer, and nominal strength of multi-walled CNTs (MWCNTs).

Figure 4 shows the model scheme for nominal strength and the relationships between the number of layers, effective strength of the outermost layer, and nominal strength of the MWCNT. The diameters shown in the legend are those of the outermost layer. In all cases, the diameter of the innermost tube was set at 1.96 nm. The results are in broad agreement with previously reported MD results [17]. For example, the nominal strength of the five-walled CNT with an outer diameter of 4.339 nm was 56.5 GPa, and hence is approximately in agreement with previous values (57.19–60.03 GPa) [17]. Therefore, the nominal strength of crosslinked MWCNTs can be estimated using the effective strength, dimensions, and number of layers. Additionally, based on Equation (9), if the inner layer contains large defects and has a lower effective strength than the outermost layer, then the effective strength of the inner layer dominates the strength of the entire CNT. However, for tensile tests of individual MWCNTs, it is difficult to evaluate which layer initiates the fracture, although kinks and discontinuous defects are observed in the inner layer of some types of MWCNTs. In view of the significance of defect densities, further studies should be carried out to evaluate the fracture mechanisms and mechanical properties.

**Figure 5:**
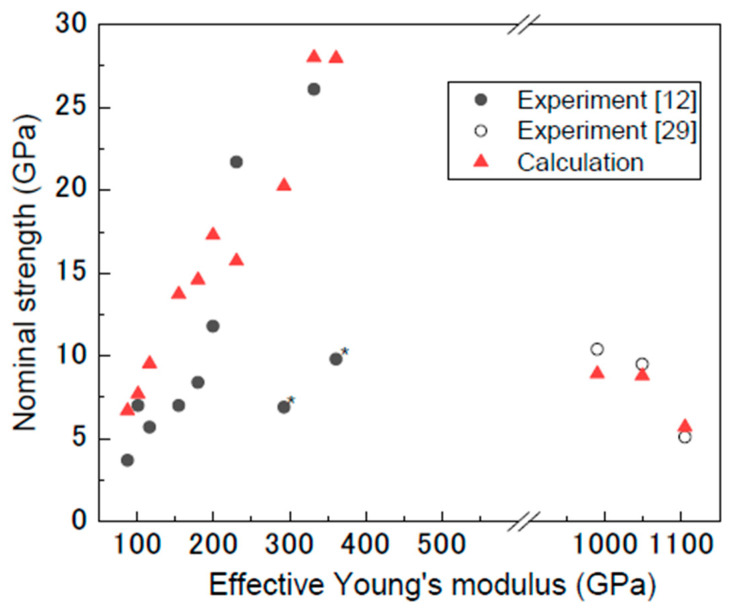
Nominal strength of CVD-grown and arc-discharge-grown MWCNTs obtained experimentally [12,29], and simulated nominal strengths obtained using Equation (7) and *σ*_ef f_= *E*_eff_/10 plotted as a function of the experimentally obtained Young’s modulus.

In addition, these results were compared with experimental data obtained from the CVD-grown and arc-discharge-grown MWCNTs [12,29]. The structure and mechanical properties of the MWCNTs and the detailed procedure for the tensile tests have been reported elsewhere [12,29]. Figure 5 shows the experimental and simulated nominal strengths against the experimentally obtained Young’s modulus. The average nominal tensile strength of the experimental CVD-grown MWCNTs was 10.8 ± 6.9 GPa [12], which is higher than that of other types of MWCNTs [22,27,33]. Note that all tested CVD-grown MWCNTs showed a clean break upon failure, whereas only the outermost layer of the arc-discharge-grown MWCNTs fractured. The simulated values were obtained using Equation (7) and $\sigma_{\mathrm{eff}}=E_{\mathrm{eff}}/10$, in which *E*_eff_ is the experimentally obtained effective Young’s modulus. The simulated nominal strengths are comparable to the experimental values, except for two points denoted by an asterisk, suggesting that for the CVD-grown MWCNTs, it is difficult to further increase the nominal strength without increasing the Young’s modulus. For these two data sets, the fracture strain was approximately 3%, which is smaller than the other data points, suggesting that premature fracture occurred due to large defects such as kinks in the nanotube layers. The effective strength was less than 30 GPa. Therefore, assuming that there are no severe defects that induce premature fracture, the pitting density of this type of CVD-grown MWCNT is above 10% (Figure 2a).

In a subsequent case study, we consider increasing the nominal strength of CNTs to 20 GPa. An effective strength of approximately 60 GPa was required for an SWCNT (chirality of (52,0)), and 40 GPa (61,0) (52,0) for a double-walled CNT, with outer diameters of 4.07 and 4.78 nm, respectively (see the square symbols shown in Figure 4). Using Figure 2, to increase the effective strength of (52,0) and (61,0) SWCNTs to 60 GPa (which corresponds to approximately 600 GPa in the effective Young’s modulus), the pitting density must be reduced to approximately 2% and 5%, respectively. The crystallinity and Young’s modulus of CNTs have been improved by thermal annealing methods [27,33,34,35,36,37] and coating (adhering) novel nanotube or graphene layers [38,39,40,41,42]. However, as shown in Figure 1, few vacancies are evident on the surfaces with a pitting density of 2–5% and, therefore, it is difficult to increase the crystallinity and effective Young’s modulus of CNTs synthesized by the CVD method to this level [22]. In addition, a decrease in the number of interlayer crosslinks may cause the fracture mode to change from a clean break mode to a sword-in-sheath failure mode [27]. In comparison, even if the effective strength of the outermost layer is 20 GPa, the nominal strength can be reached at 20 GPa for six to nine walled CNTs with outer diameters of 5.48, 6.19, 6.89, and 7.95 nm, respectively (circle symbols in Figure 4). Therefore, thin layers of CNTs may not result in an increase in the nominal strength, and desired improvements could be achieved with multilayers.

## 4. Conclusions

We presented MD simulations showing the effects of pitting on the mechanical properties of SWCNTs. The Young’s modulus decreased sharply, and approximately linearly, as the pitting density increased. Additionally, an increase in pitting density had a larger impact on the initial decrease in effective tensile strength than was seen with increasing the Young’s modulus. These trends were in good agreement with previous studies. We also proposed that it is necessary to consider the nominal strength as the index of load-bearing ability in order for the mechanical properties of CNT-reinforced composites to be designed. The nominal strength decreased with increased pitting density, and also decreased as the SWCNT diameter increased, because of the increase in the cross-sectional area of the hollow core.

In addition to the MD simulation of SWCNTs, the relationships between the number of layers, effective strength, and nominal strength of MWCNTs were discussed theoretically. The nominal strength of MWCNTs obtained from the theoretical calculations was in good agreement with results obtained from MD simulations in previous studies. Furthermore, the calculated nominal strengths are comparable to the experimental values. This suggests that for the CVD-grown MWCNTs, it is difficult to further increase the nominal strength without increasing the Young’s modulus. By combining this theoretical study and the MD simulation, the presence of extensive pitting provided a possible explanation for the low nominal strengths of MWCNTs, as well as the low modulus values reported in the fracture measurements of CVD-grown MWCNTs. A pitting density above 10% led to an effective strength range below 20 GPa. As a result, the nominal strength was also limited to approximately 20 GPa, which corresponds to values commonly observed in other studies. To improve nominal strength, the effective Young’s modulus has been increased through a post-treatment such as thermal annealing of few-walled CNTs. However, the improvements are limited. Our study has demonstrated that small improvements in the Young’s moduli of MWCNTs (number of layers, 6–9) should provide higher nominal strength values. Based on this study, we hope to develop a suitable synthesis technology, and thus increase the nominal strength of CNTs and expand their practical application.
